# A Driver’s Visual Attention Prediction Using Optical Flow

**DOI:** 10.3390/s21113722

**Published:** 2021-05-27

**Authors:** Byeongkeun Kang, Yeejin Lee

**Affiliations:** 1 Department of Electronic and IT Media Engineering, Seoul National University of Science and Technology, Seoul 01811, Korea; byeongkeun.kang@seoultech.ac.kr; 2 Department of Electrical and Information Engineering, Seoul National University of Science and Technology, Seoul 01811, Korea

**Keywords:** visual attention estimation, optical flow, driver’s perception modeling, intelligent vehicle system, convolutional neural networks

## Abstract

Motion in videos refers to the pattern of the apparent movement of objects, surfaces, and edges over image sequences caused by the relative movement between a camera and a scene. Motion, as well as scene appearance, are essential features to estimate a driver’s visual attention allocation in computer vision. However, the fact that motion can be a crucial factor in a driver’s attention estimation has not been thoroughly studied in the literature, although driver’s attention prediction models focusing on scene appearance have been well studied. Therefore, in this work, we investigate the usefulness of motion information in estimating a driver’s visual attention. To analyze the effectiveness of motion information, we develop a deep neural network framework that provides attention locations and attention levels using optical flow maps, which represent the movements of contents in videos. We validate the performance of the proposed motion-based prediction model by comparing it to the performance of the current state-of-art prediction models using RGB frames. The experimental results for a real-world dataset confirm our hypothesis that motion plays a role in prediction accuracy improvement, and there is a margin for accuracy improvement by using motion features.

## 1. Introduction

### 1.1. Motivation

There has been increasing demand for smarter and safer vehicles. Large resources in both industry and academia have been allocated for the development of vehicles with a higher level of autonomy and the advancement of human-centric artificial intelligence (AI) for autonomous vehicles [1]. While driving, human drivers keep interacting with the surrounding environment for safe vehicle control, for example, monitoring other road users and detecting traffic signals. Like human drivers, autonomous vehicles also must move in safe and predictable ways without human intervention. An essential aspect in the safe movement of such intelligent vehicles is understanding the intent of human drivers and adapting human drivers’ styles to the vehicles [1,2,3,4,5,6].

As the act of driving is based on human visual processes and attention, providing some predictions on a human driver’s intent could guide safe movement for intelligent vehicles. For instance, the predictions are about which parts of scenes are related to the driving context and how these regions correlate with the driver’s focus of attention. In computer vision, such a driver’s visual attention prediction can be formulated as a pixel-wise intensity estimation problem. An attention prediction model represents a scalar quantity at every pixel location of an image and produces the corresponding attention map that directs attentive regions [7,8,9].

Attention prediction models usually use scene appearance features of images, such as color, contrast, or orientation, to detect attention locations and levels [10,11,12,13]. In addition to these scene appearance features, psychology experiments have proved that neurons in the middle temporal visual area compute local motion contrast. Such neurons underlie the perception of motion pop-out and figure-ground segmentation, which also influences the attention allocation [14,15]. As demonstrated in the previous research studies, it is well known that motion can be a crucial feature along with visual appearance features in a driver’s attention prediction. However, the fact that motion-based features can improve prediction accuracy has been received limited attention in the literature. Therefore, in this paper, we focus on the role of motion in a driver’s attention prediction.

**Figure 1:**
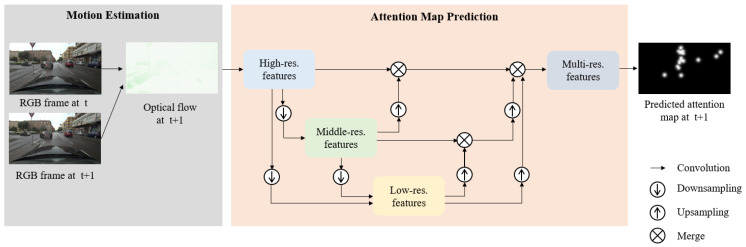
Can motion predict a driver’s attention locations? This work is motivated by the fact that attention allocations are greatly influenced by motion in dynamic scenes, not only by the visual appearance of scenes. Given an optical flow map, this work aims to predict a driver’s attention locations/levels, verifying the effectiveness of motion features in the driving context. The details of motion estimation are explained in Section 3.1, and the architecture of the proposed framework is described in Section 3.2.

We previously employed a deep neural network framework for estimating a driver’s visual attention using RGB images only [16]. We investigated that spatial features at multi-scales represent different context levels of images. Owing to the investigation, we exploited multi-scale feature extraction to improve visual attention prediction accuracy and localization performance by integrating features at different scales. Yet, we find that spatial information (e.g., color, contrast) is not the only information available, and temporal information (e.g., motion) can have a significant role in driving situation [17]. Inspiring by the fact, we develop a framework for predicting a driver’s visual attention that provides attention locations and the probability of attention levels using motion (e.g., optical flow), as described in Figure 1. Moreover, based on the framework, we further investigate visual attention prediction performance using motion-based features and demonstrate that motion plays a role in improving a driver’s attention prediction accuracy. In summary, the contributions of this work are as follows.

### 1.2. Contributions

Designing a Framework for Motion: We design a framework specifically for a driver’s visual attention prediction from spatiotemporal information. The proposed network differs from the previous driver’s attention prediction networks that focus on neighborhood information in independent frames, not considering inter-frame correlations. Specifically, the proposed framework takes optical flow maps as inputs to estimate a driver’s attention maps, unlike our previous work that takes RGB frames as inputs. As a driver’s attention to scenes requires reasonings over spatiotemporal analysis, we believe that existing driver’s attention prediction models only based on visual-appearance features are limited to the study of visual attention prediction in driving situations. For example, the attention maps predicted by the models using scene appearances tend to be biased toward the center of roads. This center-biased issue caused by stationary scene structure can be relieved by using the motions of scene contents.

Validation of Effectiveness of Motion: Based on the proposed framework, we empirically analyze the effectiveness of motion features using a real-world dataset with various scenarios. Indeed, the experimental results confirm comparability and improvement in prediction accuracy over existing methods using RGB frames only. Besides, as evidenced by the analysis, we finally draw a meaningful conclusion that motion can be a strong predictor to improve a driver’s attention prediction performance.

### 1.3. Organization

The remainder of this paper is organized as follows. In Section 2, we review the related works regarding a driver’s attention prediction based on classical attention models and convolutional neural networks for semantic segmentation. In Section 3, we provide a framework for predicting a driver’s attention to confirm our hypothesis that motion can be a strong feature in dynamic scenes, such as driving situations. Experimental results and discussion are shown in Section 4, and concluding remarks are made in Section 5.

## 2. Related Works

### 2.1. Convolutional Networks for Pixel-Wise Prediction

A driver’s attention prediction can be achieved by estimating the pixel-wise score of being attentive [16]. A driver’s attention prediction has been approached by adopting the convolutional neural networks for semantic segmentation [18] since semantic segmentation is a problem to predict the probability of being each class for each pixel [19,20,21,22,23,24]. Long et al. proposed a fully convolutional network (FCN) trained for pixels-to-pixels prediction. For spatially dense prediction, they replaced fully connected layers with convolutional layers of classification networks.

The networks inspired by these fully convolutional layers have demonstrated success at spatially dense prediction. However, pooling or strided convolution operations in the networks reduce the dimension of feature maps. Thus, the networks should upscale the reduced feature maps to produce the original input-size output prediction maps. It has been actively studied that recovering the representations to original resolution or maintaining the representations at original resolution with minimal loss of details [21,22,23,24,25,26].

In parallel to dense pixel recovery from lower dimensional outputs, multi-scale feature representation has been actively explored to improve prediction accuracy. Previous studies have confirmed that multi-scale features can capture both coarse and fine features of scenes, yielding better prediction performance [25,27,28,29]. The multi-path refinement Network (RefineNet) [30] proposed a network that exploited various levels of detail at different stages of convolutions and fused them to obtain a high-resolution prediction (a quarter of original image size) without maintaining large intermediate feature maps. The quarter resolution prediction maps were then upsampled to match the original image using bilinear interpolation. Pohlen et al. [31] combined multi-scale features using two processing streams at the full image resolution and lower resolutions undergone through pooling operations. The two streams were coupled using full-resolution residual units (FRRUs) that contain pooling, convolution, and upsampling. Zhao et al. [28] proposed the image cascade network (ICNet) that fused multi-resolution features with cascade label guidance. The network took cascade image inputs at different resolutions (i.e., low, medium, and high resolution) and adopts cascade feature fusion units that combine two resolution feature maps. The work in Reference [32] proposed densely connected atrous spatial pyramid pooling (DenseASPP) for complementing scale variation in street scenes, which requires a larger receptive field and features at various levels. The DenseASPP structure could produce a denser feature pyramid with a larger receptive field than the original atrous spatial pyramid pooling (ASPP) [25].

### 2.2. A Driver’s Attention Prediction

Toward a driver’s attention prediction, there has been extensive research on the relationship between eye movements/head positions and attention [33]. Correspondingly, a driver’s attention location was traditionally estimated from the head and eye positions or angles [34,35]. Lethaus et al. [35] demonstrated that a driver’s eye movements are strongly correlated to driving maneuvers using the gaze data acquired from a head-mounted eye-tracking system (SMI iView X^TM^ HED). The work in Reference [34] proposed a scheme that combines head pose and eye location information to improve gaze estimation. In addition, some works have tried to analyze the driver’s gaze point using motion information, such as optical flow. Wann and Land [36] visualized the optical flow from the perspective of the driver and observed future points on the expected driving path of the driver. The work in Reference [37] estimated a driver’s attention by integrating arbitrary gaze localization using optical flow. Okafuji et al. [38] used optical flow theory to figure out the driver’s gaze issue in terms of perceiving the future path of the vehicle.

As saliency models showed success at capturing human fixations [8], Pugeault and Bowden demonstrated that vision-based approaches could predict and even anticipate a driver’s behavior, using preattentive vision only [17]. Their preattentive model analyzed the association between visual attention and the random forest activation map that learned a driver’s actions in a driving context.

The recent publications of a driver’s attention prediction are based on convolutional neural networks, making significant improvements over traditional prediction models, mainly designed using hand-craft features. The work in References [18,39] proposed a computational framework using a fully convolutional neural network architecture [19,20]. Palazzi et al. estimated a driver’s focus of attention using the convolutional neural network model using RGB images, optical flow, and semantic segmentation maps [40]. Because critical driving moments are rare, Xia et al. [41] collected a new in-lab driver attention dataset, Berkeley DeepDrive Attention (BDD-A), based upon breaking evet videos. Using the dataset, they proposed the human weighted sampling (HWS) method, which uses human gaze behavior to identify crucial frames and weights them heavily during model training. The work in Reference [16] developed the high-resolution convolutional neural network framework that fuses multi-scales features, specialized for a driver’s attention prediction.

## 3. Proposed Method

As proved in the previous investigations described in Section 1 and Section 2, motion is one of the crucial factors that guide attention allocation in dynamic scenes. Indeed, the traditional spatiotemporal attention models take advantage of using appearance-based features along with motion-based features to capture the spatial and temporal characteristics in attention allocations [10,11,12,42,43,44].

Besides, a model of a driver’s attention to scenes requires reasoning over spatiotemporal saliency, moving objects, and past and possible future events [1,17]. For example, moving objects often attract more attention in driving situations like pedestrians and cyclists intended to cross the roads. Or objects in remote and occluded regions often appear to be obstacles suddenly. These examples intuitively indicate that modeling attention in independent frames is insufficient for driving tasks. It is highly desirable to model a driver’s attention taking into account temporal correlations between video frames, which provides a deeper exploration of the inter-frame information. In particular, motion-based features can give useful information about predicting attention regions in driving situations with highly complicated backgrounds and moving objects, where visual appearance features are obscured and limited.

To this end, we emphasize the role of motion in predicting a driver’s attention and present a high-resolution deep neural network that augments motion information. This work demonstrates that motion plays a role like other visual features in a driver’s attention prediction and could improve the model’s performance. The motion features are important in the sense that common driver’s attention prediction networks are focused on using a local neighborhood of image regions to predict a driver’s attention, and employing motion cues can benefit the current model.

In this section, we develop a framework to analyze the effectiveness of motion features for a driver’s attention prediction. The framework maps optical flow described in Section 3.1 to attentive locations and levels in driving situations. In particular, we investigate the performance of the motion-based framework by comparing it to the performance of the current state-of-art driver’s attention prediction network based on visual appearance features. To do this, we employ a deep neural network that learns to predict a driver’s attention, given motion features at multi-scales in Section 3.2.

### 3.1. Motion Estimation

Motion in videos is typically estimated by the optical flow, which refers to the distribution of apparent movement of brightness patterns in an image [45]. Defining f(p,t) as the intensity at the pixel location p=(x,y) of a video frame at the time *t*, optical flow is derived under the assumption that pixel intensities of an object are constant between successive frames, as follows:

(1) $$\begin{array}{c} f(p,t)=f(p+\Delta p,t+\Delta t), \end{array}$$

where Δp=(Δx,Δy), and Δt denote the spatial and temporal displacement, respectively. The purpose of optical flow is to estimate two dimensional displacement Δp=(Δx,Δy) given Δt.

Gunnar Farneback’s algorithm [46] estimates the displacement from the polynomial expansion coefficients. The algorithm approximates some neighborhoods of each pixel with a polynomial model. The model parameters are estimated from a weighted least-squares fit to the values in the neighborhood. We use the algorithm to compute dense optical flow in this work. We first convert two consecutive RGB images to grayscale images. Then, the images are downsampled to generate 0.5 scaled images and 0.25 scaled images, forming image pyramids (0.25, 0.5, 1-scaled images). The image pyramids are then used to compute optical flow. While estimating the displacement at a pixel, 5×5 pixel neighborhood regions are used with Gaussian weighting for polynomial expansion. The standard deviation of the Gaussian filter is set to 1.1. We also employ iterative displacement estimation where the number of iterations is set to 3 at each pyramid level. To suppress the effects of image noise, we average the estimated displacements over the 15×15 neighborhood region.

**Figure 2:**
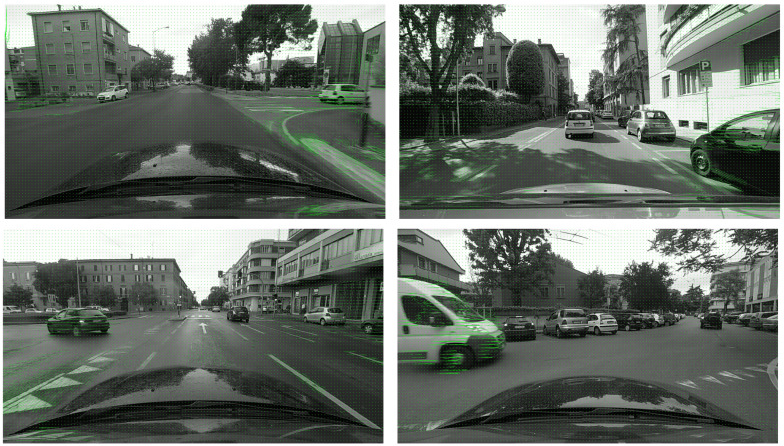
Examples of the estimated optical flow using the algorithm described in Section 3.1. The estimated dense optical flow is sampled at every 16 pixel for each dimension.

Figure 2 shows examples of the estimated optical flow. While the algorithm estimates dense optical flow, we visualized only at every 16 pixel of each dimension for better display.

### 3.2. Pixel-Wise Prediction Using Motion

Semantic segmentation is a problem that predicts the probability of being a predefined class for each pixel; thereby, a driver’s attention prediction can be solved by adopting a pixel-wise semantic segmentation. By applying semantic segmentation networks to a driver’s attention prediction, the problem is formalized as a pixel-wise intensity estimation, which value represents attention levels.

The recent works of semantic segmentation based on convolutional deep neural networks show significant improvements in prediction performance. Even though the prediction performance has been improved, in the approaches based on deep neural networks, a driver’s attention is usually predicted using the upsampled feature maps from low-resolution features. Like other deep convolutional neural network-based models, deep attention prediction networks successively reduce the spatial resolution of feature maps using pooling operations or stride convolutions. This downsampling scheme is effective in capturing large attentive areas based on coarse visual information and helps to understand global context-level visual attention. However, it comes at the cost of making the outputs coarsen and limiting the effectiveness of the prediction performance. Moreover, deconvolution often fails to recover visual features lost by the downsampling during feature extraction steps.

**Figure 3:**
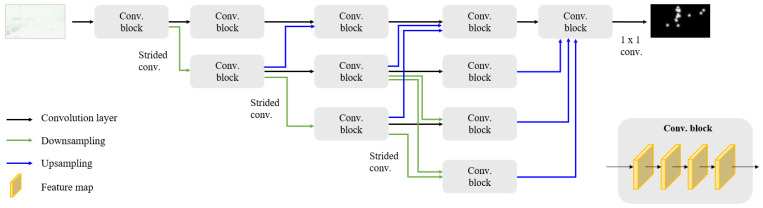
Overview of the network. The network takes optical flow maps as an input and outputs the predicted corresponding attention maps. Four sequential sub-networks represent features at different resolutions. These multi-scale features from sub-networks are fused across layers, and the high-resolution features of the original input size are maintained instead of being reduced.

To overcome the above limitations, we employ and train the fully convolutional network to be specialized at predicting a driver’s attention, inspired by the work in Reference [47], taking motion into account. The employed network takes optical flow maps as inputs, fuses multi-scale features, and defines nontrivial local-to-global and detail-to-context representations across layers, resulting in more precise prediction. As depicted in Figure 3, the network starts from a high-resolution sub-network as the first stage, gradually add high-to-low resolution sub-networks one by one to form more steps, and connect the multi-resolution sub-networks in parallel. As a result, the high-resolution motion features in the original input image resolution (in the top branch) can be maintained instead of recovering by the upsampling process, unlike typical semantic segmentation networks. Moreover, the network repeatedly fuses multi-scale motion features by exchanging the information across the parallel multi-resolution sub-networks through the entire network. This multi-scale architecture can encode both coarse global features and fine local features of a visual scene’s contents, which potentially improve prediction accuracy [25,26,27,29].

Typical semantic segmentation models output the prediction of class labels by classification formulation. In contrast to the models, the proposed model solves the regression problem of predicting a driver’s attention regions using optical flow. This is because the training goal of classification is to maximize class separation, ignoring class distances and treating all negative classes equally. It is undesirable for smoothly changing attention level assumption where the distance between current output and ground-truth determines prediction performance. Formally, the multi-scale deep neural network based on the optical flow is fine-tuned to minimize the Euclidean distance E between the vectorized version of the ground truth attention map $a\in R^{N}$ and the estimated prediction map $\hat{a}\in R^{N}$, $E=1/M\sum_{m=1}^{M}\left||{\hat{a}}^{m}-a^{m}\right|{|}_{2}^{2}$, where *N* is the number of pixels in a map, and the superscript *m* denotes the *m*-th ground truth map and the corresponding estimated map among the *M* samples in a batch.

## 4. Experimental Evaluation and Discussion

In this section, we validate the proposed driver’s attention framework using optical flow in Section 3 and discuss the experimental results using the real-world dataset below.

### 4.1. Experimental Setup

We used the dataset provided in Reference [40]. The dataset consists of 5-min-long 75 video sequences, including the scenes of landscapes, weather conditions, and daytime. In the dataset, the attention maps were captured by using a wearable eye-tracking device, *SMI ETG 2w* Glasses. To acquire the smoothed ground truth, the attention maps were further processed by accumulating the gaze point in the temporal window of k=25 frames and applying a spatial Gaussian filter with $\sigma^{2}=200$ pixels. The final attention map were then obtained by taking the maximum values of the projected gaze points over a temporal sliding window $i=\left\{-\frac{k}{2},\cdots ,0,\cdots ,\frac{k}{2}\right\}$, where *i* is the frame number. For training and inference, we followed the data separation scheme used in Reference [40]—splitting into the first 37 sequences for training and the last 37 sequences for inference. Among those sequences, the frames marked as errors by the ground truth annotations were excluded for a thorough study of prediction performance without any secondary effects. The frames collected when the speed of the vehicle was zero were also excluded for training. This is because a driver is usually inattentive to driving-related events when the vehicle is not moving [18]. For each sequence, we set Δt in (1) to 1. The optical flow from the second frame was applied for the first frame because there is no previous frame of the first frame.

To guarantee prediction performance, the implementation of the proposed network in Section 3.2 follows the practice of state-of-art models in References [26,48,49,50]. The proposed network was pretrained using the ImageNet classification dataset [51] with a weight decay of 0.0001 and a momentum of 0.9 [50]. Then, the model was trained for a driver’s attention prediction task using the dataset described above. For training, we followed the training strategy described in Reference [26] and used the stochastic gradient descent (SGD) optimization with the momentum of 0.9 and the weight decay of 0.0005. The network was first fine-tuned for 59,000 iterations, where the initial learning rate was 0.01, and each batch takes 12 optical flow maps. Then, it was fine-tuned for 26,000 iterations with the initial learning rate was 0.001. The poly learning rate policy [27] with the power of 0.9 is used for dropping the learning rate.

We considered three driver’s visual attention prediction strategies: a baseline model, deep neural network-based models, and traditional saliency detection models. It is well observed that a naive attention estimation at the center of an image could yield better inference than other saliency models [16,17,18,39,40]. Due to this reason, we considered “baseline” attention prediction by averaging ground-truth attention maps over all training sequences. For deep neural network-based models, we compared with the performance of the state-of-the-art networks using the dataset described above—the network in Reference [18] using RGB frames, the network in Reference [41] using RGB frames, the network in Reference [40] using RGB frames, optical flow maps, and segmentation maps, and the network in Reference [16] using RGB frames. In addition, we compared attention prediction performance with the traditional well-known saliency estimation algorithms: the model in Reference [8] and graph-based visual saliency in Reference [52].

### 4.2. Results and Analysis

Quantitative Analysis: For quantitative evaluation, we assessed how well the intensity at each location predicted a driver’s focus of attention. To assess attention prediction, we used a standard metric, Pearson correlation coefficient (*r*) [53,54] between the vectorized version of the normalized ground truth attention map $a^{\prime }=\left(a-\bar{a}\right)/\sigma_{a}$ and the normalized estimated prediction map $\hat{a^{\prime }}=\left(\hat{a}-\bar{\hat{a}}\right)/\sigma_{\hat{a}}$ [16,18,40], where $\bar{a}$ and $\bar{\hat{a}}$ are the means of *a* and $\hat{a}$; and $\sigma_{a}$ and $\sigma_{\hat{a}}$ the standard deviations of *a* and $\hat{a}$, respectively.

We report the attention prediction performance in Table 1 in terms of the average of the Pearson correlation coefficient over all test frames. Note that the correlation coefficient of the model in Reference [41] was measured from the network that trained using the selected 200 ten-second long video clips, and the model was then fine-tuned with their own dataset proposed in Reference [41]. The deep neural network-based models outperformed the traditional saliency models. The traditional models had very weak correlations with a driver’s attention and appeared to carry less predictiveness of the driver’s visual attention. This was expected because the traditional models estimate attentive regions only based on discernible visual appearance.

The highest performing was reached by the model in Reference [16] using RGB frames. The prediction performance improved by about 25.6% for the model relative to the baseline model. Though all correlation coefficients for other deep neural network-based models were decent, the second-highest correlation coefficient was observed for the proposed framework using optical flow map and improved by about 23.4% relative to the baseline model. This explicitly implies that motion can improve the prediction performance by leveraging visual features. Similarly, the model in Reference [40] using both RGB frames, optical flow, and segmentation maps improved attention prediction performance by 19.1% relative to the baseline model. This is consistent with the assumption that attention prediction implicitly takes advantage of the visual appearance information, as well as motion information. More importantly, the average correlation coefficient value of the proposed network indicates the margin of improvement in a driver’s attention prediction when using both visual and motion features in multi-scale, as evidenced by other average correlation coefficient values in Table 1.

In order to examine the effectiveness of motion, we created a test to compare the performance of the proposed model using optical flow against the current state-of-art model using RGB frames only for the frames captured in the velocity faster than 5 km/h or 30 km/h. In the test, we counted the number of frames that the proposed model outperforms the current state-of-art model among the test frames. The results in percentage are summarized in Table 2. As demonstrated in the proportions, the proposed model comparably performed well over the current-state-of model when scene motions were observed. This indicates that motion features played a role as the vehicles moved and the scene movements become more noticeable. Indeed, the average correlation coefficient of the proposed model reached (or even exceed slightly) that of the current state-of-art model for the frames with movements, although the average correlation coefficient of the proposed model is slightly lower than that of the current state-of-art model over all test frames in Table 1. The average correlation coefficients are approximately 0.61 and 0.63 for both the proposed model and the current state-of-art model over the test frames with the velocity faster than 5 km/h and 30 km/h, respectively.

**Figure 4:**
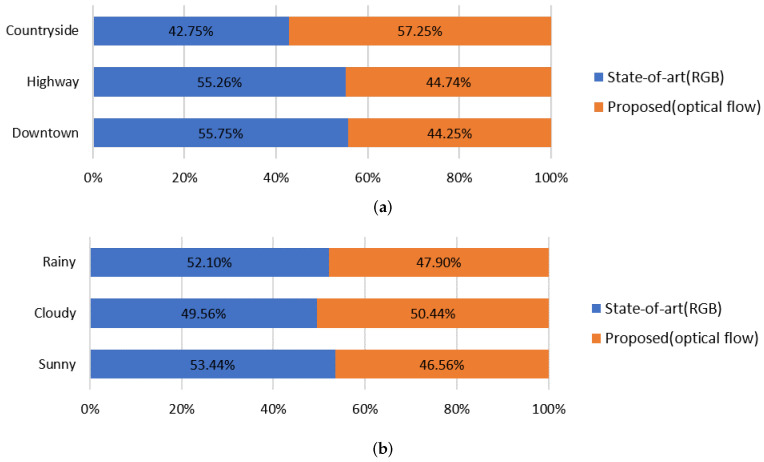
Analysis of the proportion of the frames each model performs better with different environmental conditions. (**a**) Locations (countryside, highway, and downtown). The average velocity of the scenes of countryside, highway, and downtown are 50.57 km/h, 78.95 km/h, and 26.90 km/h, respectively. (**b**) Weather conditions (rainy, cloudy, and sunny). The average velocity of the scenes of rainy, cloudy, and sunny days are 47.51 km/h, 50.35 km/h, and 51.09 km/h, respectively.

We also studied how many frames the proposed model outperforms the current state-of-art model [16] under different environmental conditions (location and weather) in percentage. As shown in the graph of Figure 4a, the proposed model performed well in the countryside scenes, where visual appearance features were less distinct than in the downtown scenes. In the downtown frames, due to the low speed of vehicles, the effect of optical flow was also likely to be less discernible. Comparing to highway scenes, as a vehicle’s velocity increases, a driver focuses on the center of the road and tends not to distract by irrelevant surrounding objects. The current state-of-art network using RGB frames well learned this situation, where typical driving datasets tend to bias toward vanishing points of the scenes. Moreover, in the graph of Figure 4b, the current state-of-art network performed well in the frames captured on sunny or rainy days. This is presumably because visual appearance features are more likely to be discernible on sunny days. And subtle movements of the environment could hinder accurate attention locations on rainy days.

The analysis in Table 2 and Figure 4 demonstrates that different types of features play different roles in a driver’s attention prediction. Based on the analysis, we conclude that spatiotemporal information can enhance driver’s attention prediction accuracy when leveraging current prediction models.

**Figure 5:**
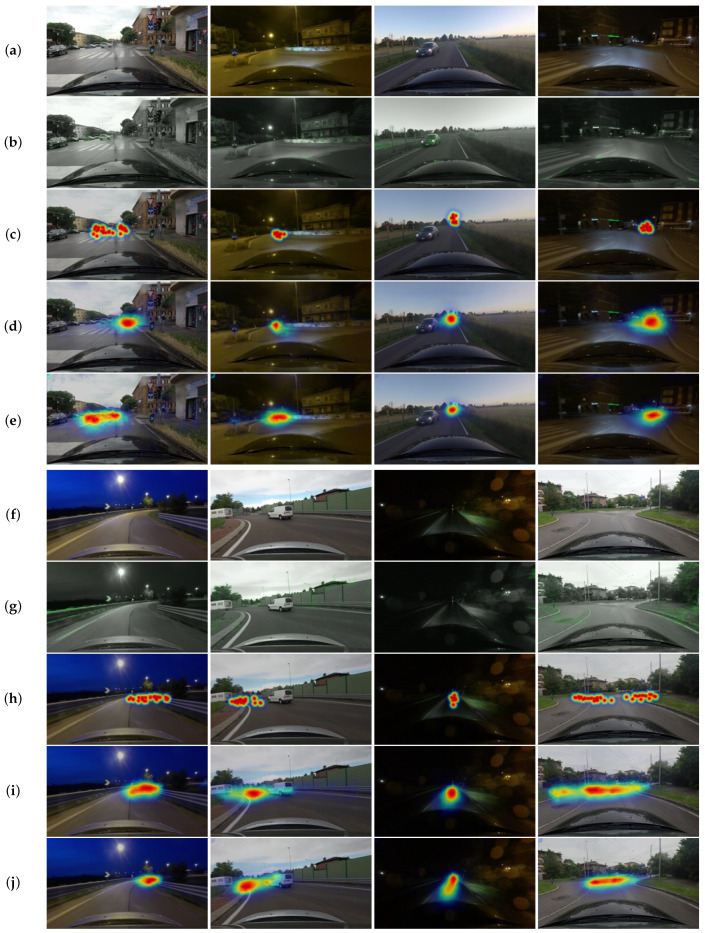
Examples of predicted attention maps. The output attention maps overlay with the input images and scale to RGB components. (**a**) Input Image. (**b**) Optical flow map. (**c**) Ground-truth attention map. (**d**) Attention map of the state-of-art model using RGB frames. (**e**) Attention map of the proposed model using optical flow. (**f**) Input Image. (**g**) Optical flow map. (**h**) Ground-truth attention map. (**i**) Attention map of the state-of-art model using RGB frames. (**j**) Attention map of the proposed model using optical flow. The proposed model using optical flow can also detect contextual and pixel-wise labels consistently and accurately, comparable to the model using RGB frames.

Qualitative Analysis: Figure 5 demonstrates the visual comparisons of the proposed model using optical flow maps and to the current state-art-of deep neural network-based model using RGB frames. Overall, both models could provide acceptable localization performance and prediction performance, as expected. The model using optical flow maps was able to predict contextual and pixel-wise labels consistently and accurately, comparable to the state-art-of model using RGB frames, as shown in the examples of the estimated attention maps. This also confirms our observation that motion can be a good predictor for a driver’s visual attention.

**Figure 6:**
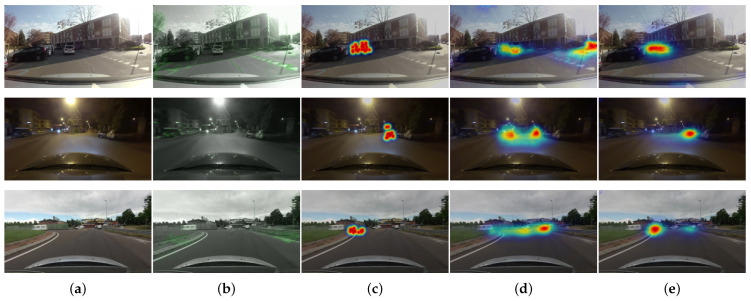
Visual analysis of failure cases using RGB frames. (**a**) Input Image. (**b**) Optical flow map. (**c**) Ground-truth attention map. (**d**) Attention map of the state-or-art model using RGB frames. (**e**) Attention map of the proposed model using optical flow. These failure cases imply that the networks for a driver’s attention prediction can easily learn attention regions nearby vanishing points.

**Figure 7:**
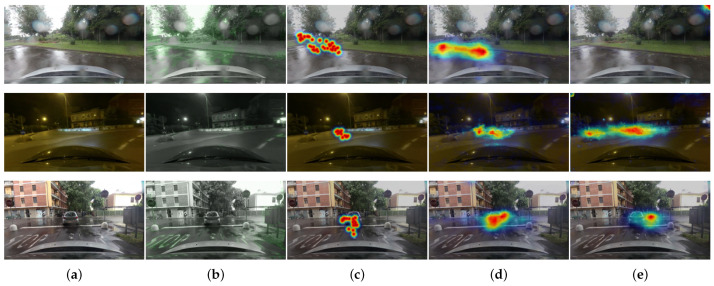
Visual analysis of failure cases using optical flow maps. (**a**) Input Image. (**b**) Optical flow map. (**c**) Ground-truth attention map. (**d**) Attention map of the state-or-art model using RGB frames. (**e**) Attention map of the proposed model using optical flow. These failure cases imply that the proposed network can also learn unsteady small variations in the surrounding environment.

We also show failure cases of the current state-of-art model using RGB frames in Figure 6. Some failure cases show situations where the estimated attention regions are toward vanishing points. This is a fundamental difficulty when predicting a driver’s visual attention. Drivers tend to stare at the vanishing points or center of the roads; therefore, such sorts of examples are abundant in the driving dataset. Consequently, prediction models could easily learn center-biased attention regions. However, as shown in the examples estimated by the proposed model using optical flow, the center bias effect (nearby the vanishing points) decreases with dynamic stimuli. This sufficiently indicates that motion information can be reasonably assumed to contribute to salient region detection since the pixels which change in the optical flow field often attract more attention. In contrast, the temporal saliency is not perfectly equal to the amplitude of all the motion. For example, subtle motions from unsteady small disturbance or illumination changes in the environment can be learned as non-trivial features by the network, negatively impacting prediction accuracy. The examples of the top and bottom rows of Figure 7 shows that subtle movement of water drops can make the network confused to predict salient regions. And the examples of the middle row of Figure 7 shows that the network could detect the areas visually obtruded by illumination changes as salient regions. The observations demonstrate that irregular patterns could impede the network using the optical flow from estimating accurate attention regions, resulting in poor prediction accuracy.

Nevertheless, as indicated in the examples of Figure 6 and Figure 7, there is a margin for improving attention prediction accuracy and localization performance when leveraging motion features with visual appearance features.

## 5. Conclusions

This paper developed a framework to analyze the drivers’ attention prediction performance based on motion features. To investigate the effectiveness of motion features, we proposed a multi-scale framework that augments optical flow to improve the prediction performance. The usefulness of motion features was verified by comparing the prediction accuracy of the proposed network and the current state-of-art networks using RGB frames. As a result, the comparisons confirm that the prediction accuracy of the attention maps generated by the proposed network was comparable to that of the current state-of-art network. This fact sufficiently supports our assumption that motion features are important in the sense that motion cues pushing drivers’ attention can benefit the current model with visual features only. Further investigation of training using the fused visual appearance and motion features will be a subject of future study. We hope that this study will motivate further developments in spatiotemporal drivers’ attention prediction and essential for real-world applications.

## Figures and Tables

**Table 1:** Performance of the proposed model using optical flow against other models in terms of the Pearson correlation coefficient.

Model	Average Correlation Coefficient
Baseline	0.47
Itti et al. [8]	0.16
Harel et al. [52]	0.20
Xia et al. [41]	0.51
Tawari et al. [18]	0.55
Palazzi et al. [40]	0.56
Kang et al. [16]	0.59
Proposed (Optical Flow)	0.58

**Table 2:** The number of frames that the proposed model outperforms the current state-of-art model among the frames with the velocity higher than 5 km/h or 30 km/h (i.e., >5 km/h or >30 km/h).

	State-Of-Art (RGB) [16]	Proposed (Optical Flow)
>5 km/h	49.5 %	50.5 %
>30 km/h	48.9 %	51.1 %
